# Salvaged allogeneic hematopoietic stem cell transplantation for pediatric chemotherapy refractory acute leukemia

**DOI:** 10.18632/oncotarget.22809

**Published:** 2017-12-01

**Authors:** Jingbo Wang, Lei Yuan, Haoyu Cheng, Xinhong Fei, Yumin Yin, Jiangying Gu, Song Xue, Junbao He, Fan Yang, Xiaocan Wang, Yixin Yang, Weijie Zhang

**Affiliations:** ^1^Department of Hematology, China Aerospace Central Hospital, Beijing, China

**Keywords:** hematopoietic stem cell transplantation, pediatric, acute leukemia, primary refractory, relapsed refractory

## Abstract

There is an ongoing debate concerning the performance of salvaged allogeneic hematopoietic stem cell transplantation (allo-HSCT) in pediatric patients with acute refractory leukemia, in whom the prognosis is quite dismal. Few studies have ever been conducted on this subject. This may be partly due to missed opportunities by majority of the patients in such situations. To investigate the feasibility, evaluate the efficiency, and identify the prognostic factors of allo-HSCT in this sub-setting, the authors performed a single institution-based retrospective analysis. A total of 44 patients, of whom 28 had acute myeloid leukemia (AML), 13 had acute lymphocytic leukemia (ALL), and 3 had mixed phenotype leukemia (MPL), were enrolled in this study. With a median follow-up of 19 months, the estimated 2-year overall survival (OS) and progression free survival (PFS) were 34.3% (95% CI, 17.9–51.4%) and 33.6% (95% CI, 18.0–50.1%), respectively. The estimated 2-year incidence rates of relapse and non-relapse mortality (NRM) were 43.8% (95% CI 26.4–60.0%) and 19.6% (95% CI 9.1–32.9%), respectively. The estimated 100-day cumulative incidence of acute graft versus host disease (aGvHD) was 43.6% (95% CI 28.7–57.5%), and the 1-year cumulative incidence of chronic GvHD (cGvHD) was 45.5% (95% CI 30.5–59.3%). Compared with the previous studies, the multivariate analysis in this study additionally identified that female donors and cGvHD were associated with lower relapse and better PFS and OS. Male recipients, age younger than 10 years, a diagnosis of ALL, and the intermediate-adverse cytogenetic risk group were associated with increased relapse. On the contrary, extramedullary disease (EMD) and aGvHD were only linked to worse PFS. These data suggested that although only one-third of the patients would obtain PFS over 2 years, salvaged allo-HSCT is still the most reliable and best therapeutic strategy for refractory pediatric acute leukemia. If probable, choosing a female donor, better management of aGvHD, and induction of cGvHD promotes patient survival.

## INTRODUCTION

Significant breakthroughs have been achieved in the clinical management of acute leukemia in the pediatric population, resulting in a near 90% and approximate 60% cure rates in the acute lymphoblastic leukemia (ALL) and the acute myeloid leukemia (AML), respectively [1–5]. The prognosis of patients who experienced relapsed refractory (R/Ref) or primary refractory (P/Ref), however, remains a therapeutic challenge even in the current era with increasing numbers of novel agent-based clinical trials [6, 7]. Nearly 15% of all pediatric ALL eventually relapse, which is similar to the incidence of childhood AML cases [1]. Among these children, only less than 50% could be cured with intensified chemotherapy and allogeneic hematopoietic stem cell transplantation (allo-HSCT) [8–11]. Thus, relapsed ALL is considered as the fourth most common pediatric malignancy in the world. Meanwhile, a prevalence of 5% to 10% *de novo* and 23% of relapsed pediatric AML exhibit the characteristic of multi-drug resistance and refractory disease [12]. The overall survival (OS) rates of this extreme subgroup of patients were only 22% in the P/Ref and 14% in the R/Ref settings [12]. With limited studies, widely varying survival rates of 0% to 35% and a non-relapse-related mortality (NRM) rate as high as 40% have been reported from four studies involving less than 200 subjects [8–11].

The overall long-term survival rates of these patients are dismal, even with the advent of current novel agents [13, 14]. Although increasingly new drugs, monoclonal antibody-based therapies, and adoptive immune-therapeutic strategies have successfully improved patient’s remission rates, a longer duration of remission has been difficult to obtain [15, 16]. Thus, it is more reasonable to perform allo-HSCT bridged with the abovementioned novel strategies [17]. However, currently, there is no professional consensus or guideline from the limited clinical studies to determine whether pediatric patients with P/Ref or R/Ref acute leukemia would benefit more from receiving a salvaged allo-HSCT than from an intensified chemotherapy [18–20]. Due to the ongoing debate about the necessity of salvaged allo-HSCT for pediatric P/Ref and R/Ref acute leukemia, only a few studies have specifically examined the efficiency of allo-HSCT for those subjects. Moreover, most of the previous reports failed to identify the prognostic factors of allo-HSCT in such situation [9, 21]. Allo-HSCT was performed in 200 patients with childhood AML as reported in the present literature, including two prospective studies. As the long-term survival following allo-HSCT is less than 20%, most of the institutions do not recommend this therapy in patients with AML with active disease and in those with over 25% leukemic blasts in the bone marrow (BM) [8]. In addition, the results from the European Blood and Marrow Transplantation Registry revealed that the event-free survival (EFS) among the 127 children who received haplo-identical HSCT not in remission was 0% [22]. Thus, pediatric ALL not in remission is a contraindication of allo-HSCT.

On the contrary, several studies have reported the success in salvaging adult P/Ref and R/Ref acute leukemia with allo-HSCT [23, 24]. Most of these have incorporated with pre-HSCT intensified chemotherapy, rapid withdrawal of immunosuppressive therapy (IST), or minimal residual disease (MRD)-guided donor lymphocyte infusion (DLI) [25]. Therefore, with the improvement of allo-HSCT in treating adult patients in similar situation, the outcomes of allo-HSCT in pediatric patients must be investigated. In this study, we report the outcome of allo-HSCT for pediatric P/Ref and R/Ref acute leukemia and analyze the feasibility of using this treatment as an alternative salvage therapy for these types of patients.

## RESULTS

### HSCT procedure

All 44 patients (100%) received myeloablative (MAC) conditioning HSCT (Table 1). Specifically, 31 patients (17 AML, 12 ALL, and 2 MPAL) had TBI-based conditioning, whereas the remaining 13 (11 AML. 1 ALL and 1 MPAL) received Bu-based conditioning regimen. FLAG chemotherapy and CLAG chemotherapy were used in 24 and 8 patients, respectively. Idarubicin was used in 26 patients along with cytoreduction chemotherapy (*N* = 20) or prior to HSCT conditioning for those did not receive cytoreduction chemotherapy (*N* = 6). HLA-matched sibling and haplo-identical transplantation were available in 7 and 37 patients, respectively. All patients received BM and PBSCs from the same donor as grafts. Moreover, 28 patients received additional UCB infusion to enhance engraftment, whereas 16 patients did not due to all sorts of contraindication including hypertension (*N* = 6), fever (*N* = 4), low oxygen saturation (*N* = 4), allergic reaction (*N* = 1), and vital organ immune impairment (*N* = 1).

**Table 1:** Characteristic of HSCT

Variables	
Donor characteristics, n (%)	
HLA-haplo-identical	37 (84.1)
HLA-identical sibling	7 (15.9)
Source of stem cells	
PB+BM	44 (100)
Cell dose Cell dose (MNC cells/Kg)	
MNC (108 cells/Kg) (range)	8.96 (7.98–24.40)
CD34+ (106 cells/Kg) (range)	3.34 (0.80–7.76)
Gender (donor/recipient), n (%)	
M/M	20 (45.5)
F/M	11 (25.0)
M/F	9 (20.5)
F/F	4 (9.0)
UCB infusion prior to HSCT, n (%)	
Yes	28 (63.6)
No	16 (36.4)
UCB HLA matching, n (%)	
6/6	3/28 (10.7)
5/6	16/28 (57.1)
4/6	9/28 (32.1)
GvHD prophylaxis, n (%)	
CSA+MMF+MTX	44 (100)
TBI-based conditioning, n (%)	
Yes	31 (70.5)
No	13 (29.5)
Intensified chemotherapy in conditioning, n (%)	
Null	12 (27.3)
FLAG	24 (54.5)
CLAG	8 (18.2)
DLI, n (%)	
Yes	15 (34.1)
No	29 (65.9)

Abbreviations: UCB, umbilical cord blood; TBI, total body irradiation; CLAG, cladribine, cytarabine, granulocyte colony stimulating factor; FLAG, fludarabine, cytarabine, granulocyte colony stimulating factor; DLI, donor lymphocyte infusion; M, male; F, female; GvHD, graft versus host disease; CSA, cyclosporine A; MMF, mycophenolate mofetil; MTX, methotrexate; BM, bone marrow; PB, peripheral blood; MNC, mononucleated cell

### Engraftment and GvHD, virus activation

**Figure 1:**
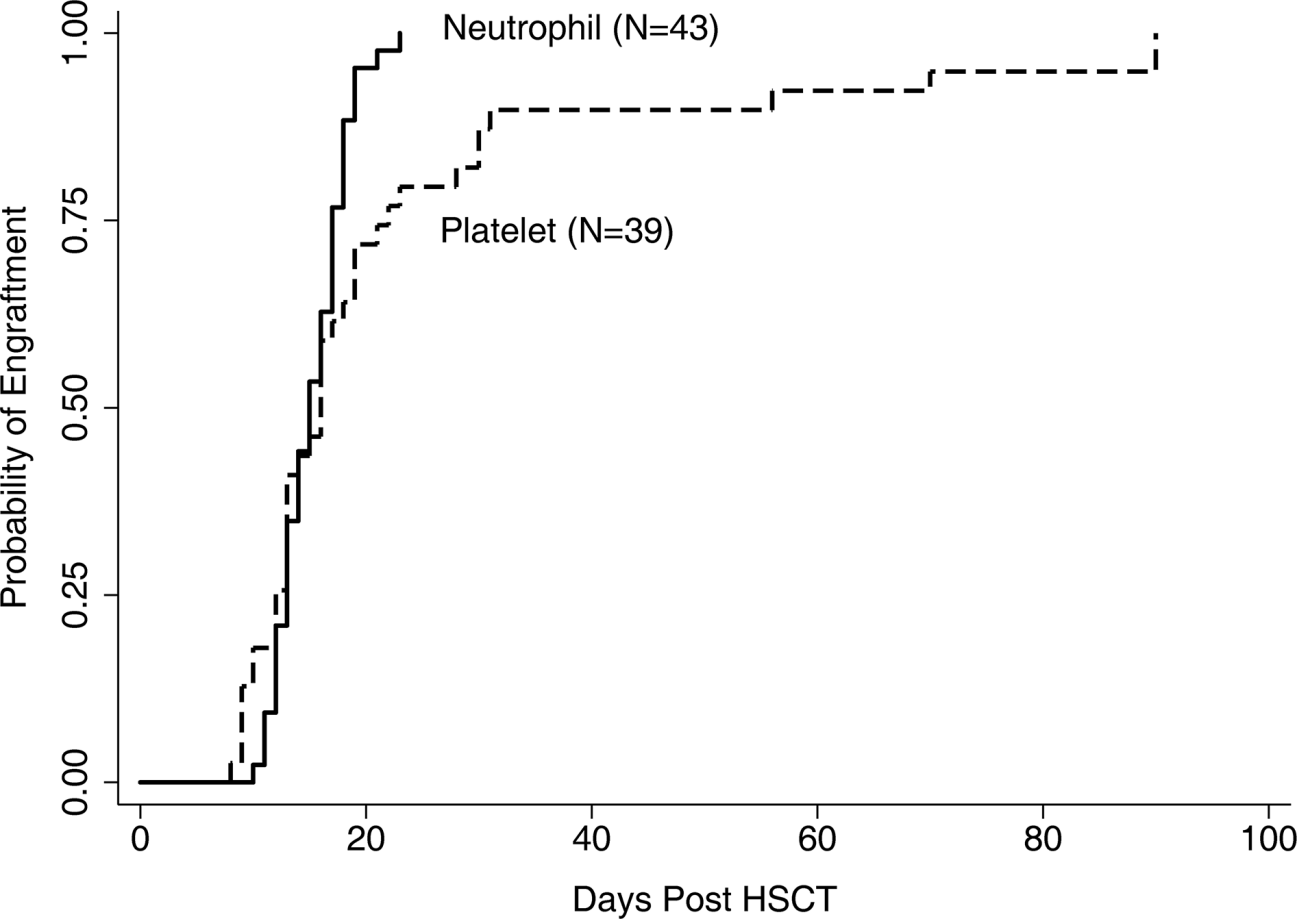
Engraftment of neutrophil and platelet One patients failed to achieve neutrophil and platelet engraftment and another 4 experienced failure of platelet engraftment till 56 days after transplantation.

Hematological recovery was almost uneventful. All except one patient failed to engraft within the first 2 months after HSCT and eventually died of relapse 5 months following HSCT. All of the remaining patients successfully obtained neutrophil engraftment, but four of them experienced failure of platelet engraftment. Among all the evaluable patients, the median time of neutrophil engraftment was 16 days (range 10–23 days), while that of the platelet engraftment was 19 days (range 8–90 days) (Figure 1). All 44 patients achieved a complete donor chimerism on day +28 with CR.

**Figure 2:**
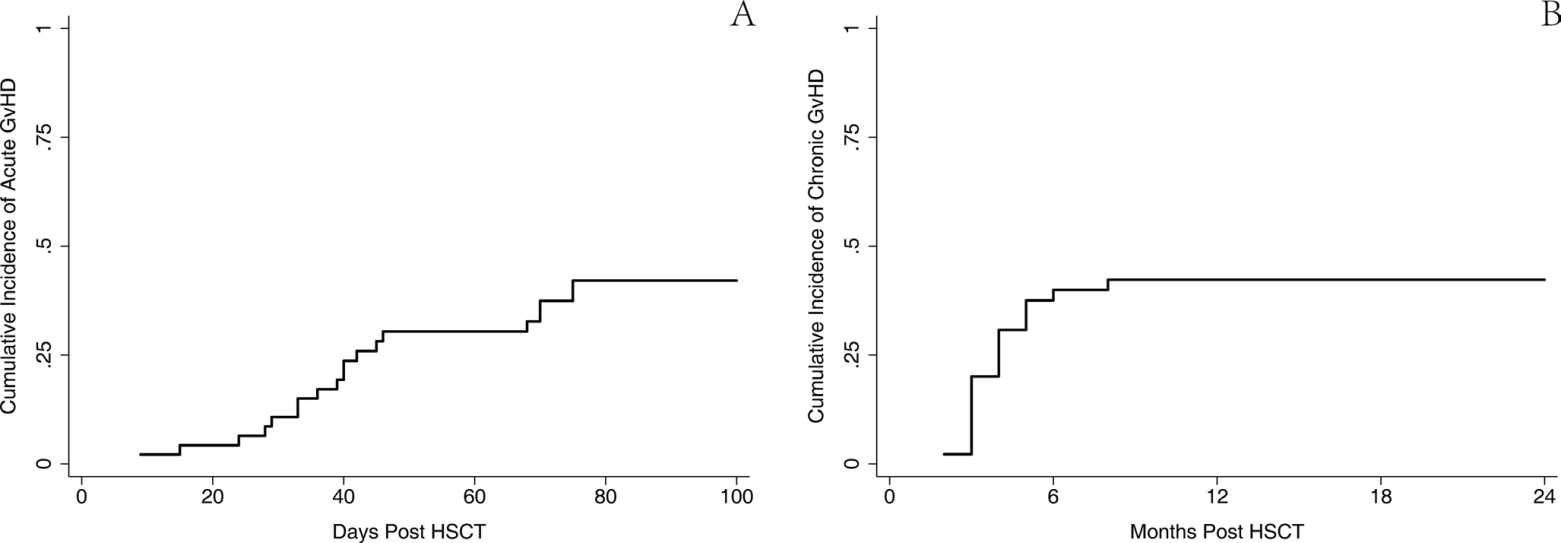
Cumulative incidence of acute and chronic GvHD (**A**) The estimated 100-day cumulative incidence of aGvHD was 43.6% (95% CI 28.7-57.5%). (**B**) 1-year cumulative incidence of cGvHD was 45.5% (95% CI 30.5–59.3%).

The incidence of GvHD was relatively higher than those reported in other studies. aGvHD within the first 100 days was reported in 19 patients (43.2%). A 3–4 degree aGvHD was seen in 7 patients (15.9%). The estimated 100-day cumulative incidence of aGvHD was 43.6% (95% CI, 28.7–57.5%). A total of 20 patients (45.5%) experienced cGvHD, including 12 cases (27.3%) with extensive cGvHD. The estimated 1-year cumulative incidence of cGvHD was 45.5% (95% CI 30.5–59.3%) (Figure 2). Nonfatal EBV reactivation occurred within 100 days in four patients. Only one patient died of CMV-related encephalitis on day 58 after HSCT.

### NRM, relapse, and cause of death

**Figure 3:**
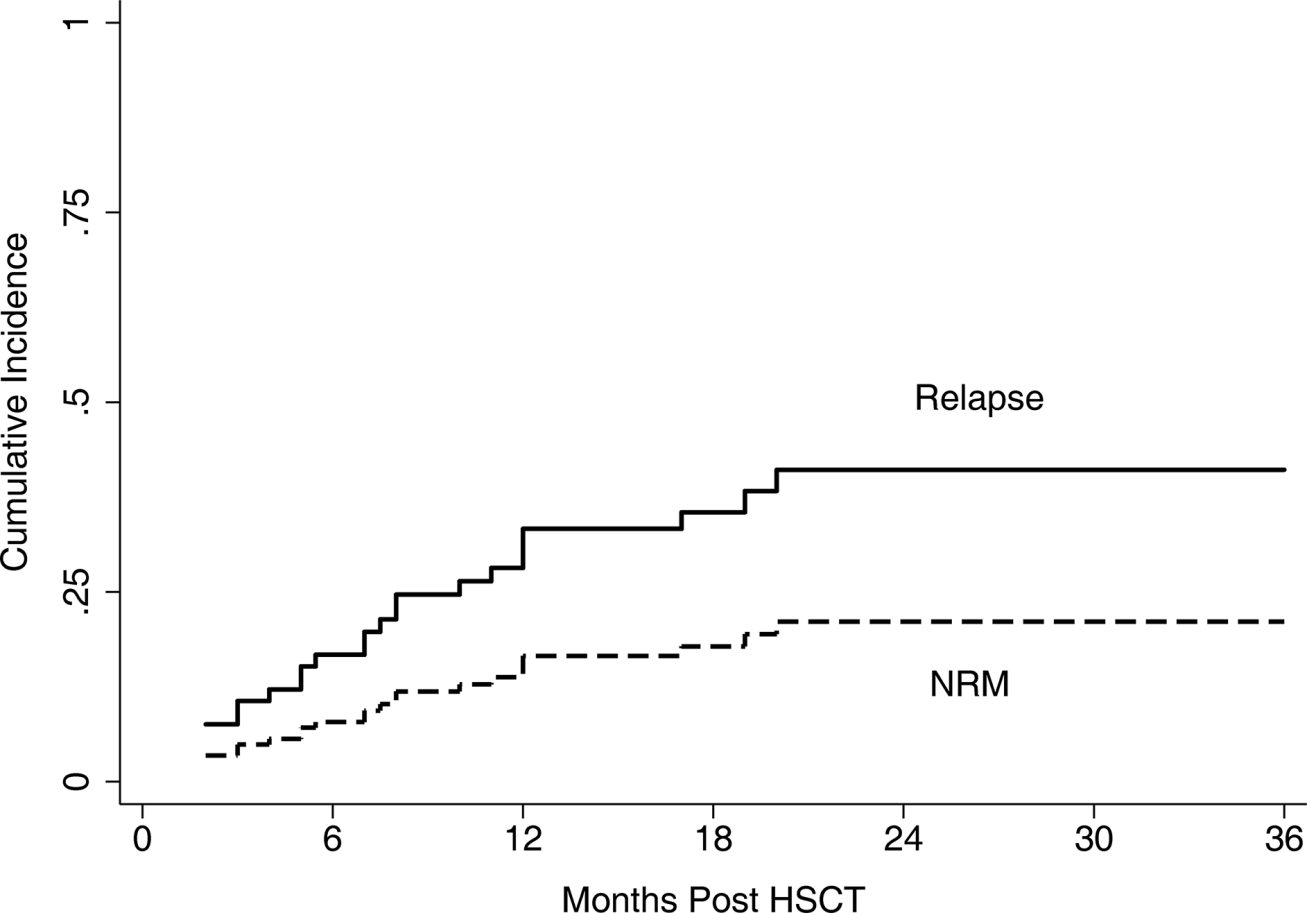
Cumulative incidence of relapse and NRM The estimated 2-year incidence of relapse and NRM was 43.8% (95% CI 26.4–60.0%) and 19.6% (95% CI 9.1–32.9%), respectively.

Figure 3 showed the cumulative incidence curve of relapse and NRM. In brief, disease recurrence remained the major reason of HSCT failure; however, NRM was not increased significantly compared with allo-HSCT in regular patients. One patient failed to engraft and died of relapse 5 months after transplantation. Another 15 relapses occurred, and all died within the first 2 years. Specifically, 8 patients died from NRM, 3 from bronchiolitis obliterans (BO), 2 from infection, 2 from diffuse alveolar hemorrhage (DAH), and 1 from viral encephalitis. The estimated 2-year incidence rates of relapse and NRM were 43.8% (95% CI, 26.4–60.0%) and 19.6% (95% CI, 9.1–32.9%), respectively.

### OS and PFS

**Figure 4:**
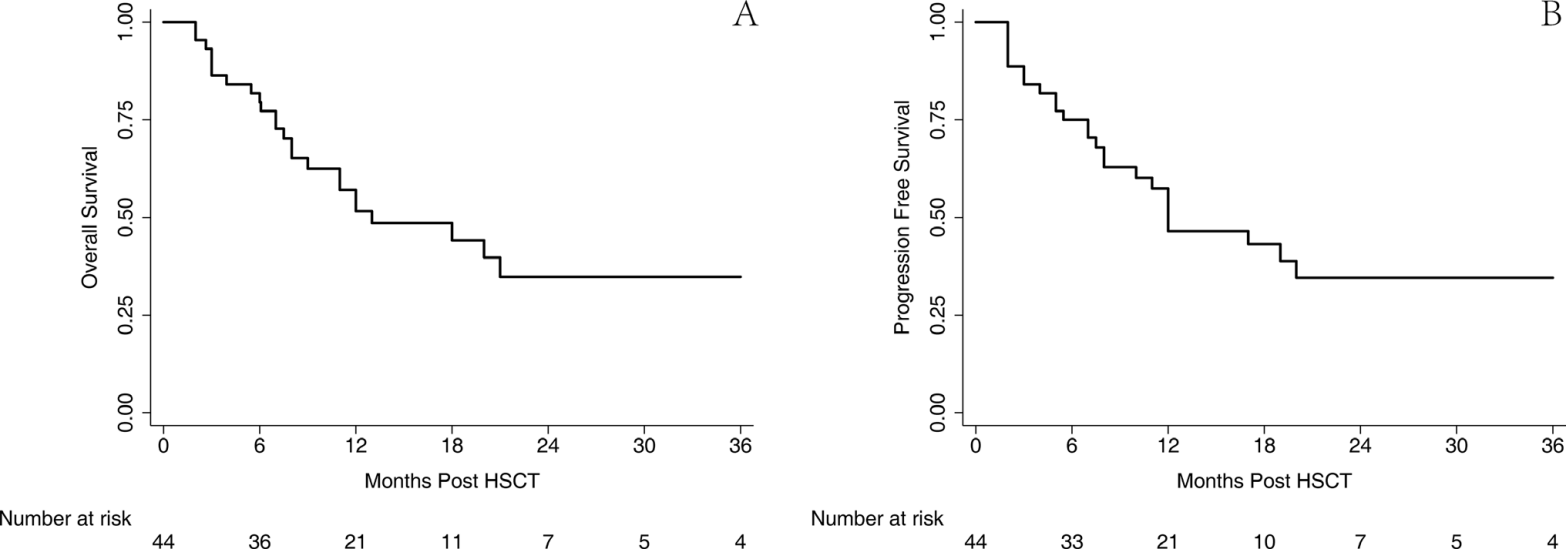
OS and PFS of the entire cohort The estimated 2-year OS (**A**) and PFS (**B**) was 34.3% (95% CI 17.9–51.4%) and 33.6% (95% CI 18.0–50.1%), respectively.

**Figure 5:**
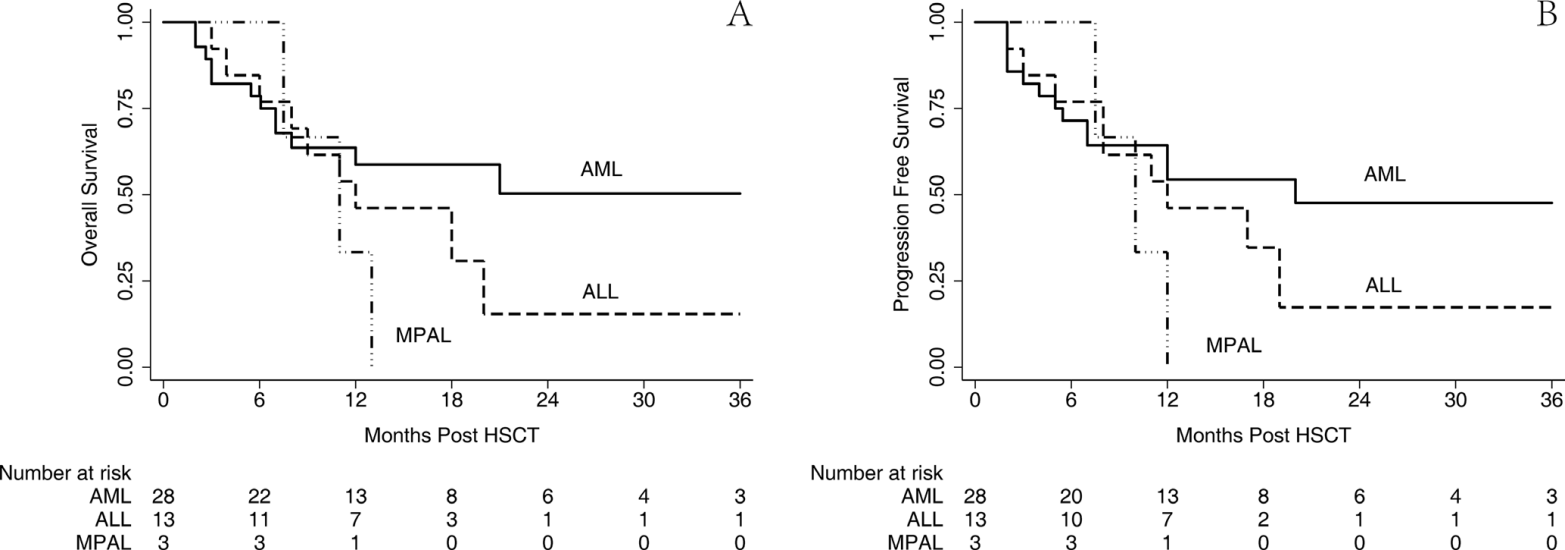
OS and PFS stratified by diagnosis The estimated 2-year OS for AML, ALL and MPL was 49.5% (95% CI 26.3–69.2%), 15.2% (95% CI 0.9–46.7%) and 0.0% (95% CI 0.0-0.0%) respectively (**A**) and 2-year PFS for AML, ALL and MPL was 46.7% (95% CI 24.8-66.0%), 16.3% (95% CI 1.1–48.1%) and 0.0% (95% CI 0.0–0.0%), respectively (**B**).

The outcome of survival was comparable with that from previous published reports although the follow-up duration was relatively short. Altogether, 20 patients survived in the CR until the endpoint of this retrospective analysis, as shown in Figure 4; the estimated 2-year OS and PFS were 34.3% (95% CI 17.9–51.4%) and 33.6% (95% CI 18.0–50.1%), respectively, with a median follow-up of 19 months. Then, this result was stratified by the disease group, which is illustrated in Figure 5; the estimated 2-year OS for AML, ALL, and MPL was 49.5% (95% CI, 26.3–69.2%), 15.2% (95% CI, 0.9–46.7%), and 0.0% (95% CI, 0.0–0.0%), respectively, and 2-year PFS for AML, ALL, and MPL was 46.7% (95% CI, 24.8–66.0%), 16.3% (95% CI, 1.1–48.1%), and 0.0% (95% CI, 0.0–0.0%), respectively.

### Survival analysis of risk group, disease status prior to HSCT, and GvHD

**Figure 6:**
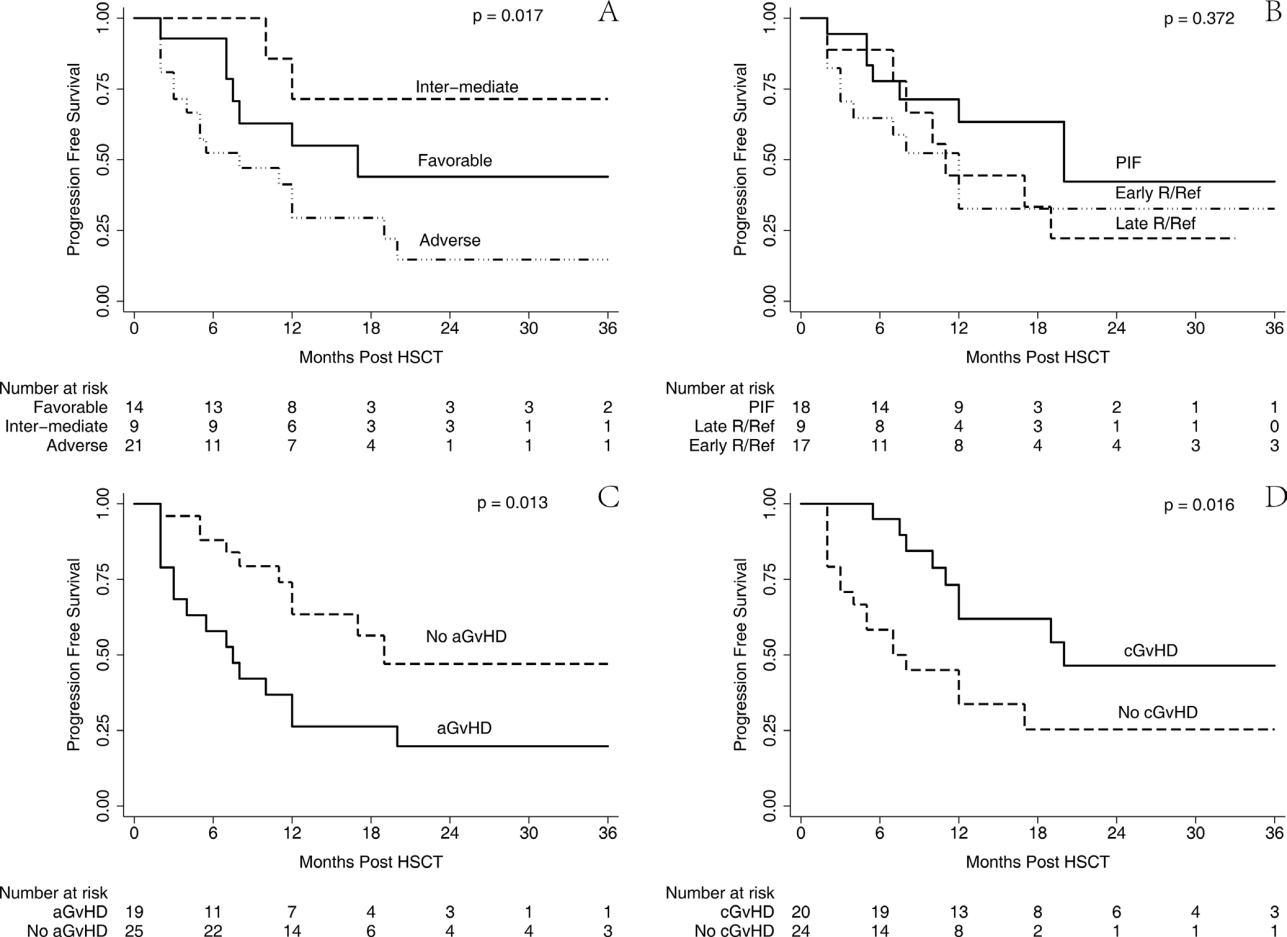
PFS stratified by risk group, disease status, aGvHD and cGvHD Univariate analysis by Log-Rank test and Kaplan-Meier survival curve for PFS stratified by risk group (**A**), disease status at transplantation (**B**), acute GvHD (**C**) and chronic GvHD (**D**), respectively.

Among the study cohorts, we analyzed the impact of risk groups, disease status prior to HSCT, and acute and chronic GvHD on patients’ survival. The estimated 2-year PFS in patients with favorable risk was 42.3% (95% CI, 14.3–68.4%), inter-mediate risk was 71.4% (95% CI, 25.8–92.0), and adverse risk group was 13.2% (95% CI, 1.9–35.5%), (*p* = 0.017) (Figure 6A). The estimated 2-year PFS in patients with P/Ref was 42.2% (95% CI, 9.0–73.5%), early R/Ref was 32.5% (95% CI, 12.1–55.0), and late R/Ref was 22.2% (95% CI 3.4–51.3%), (*p* = 0.372) (Figure 6B). Patients without aGvHD had a significantly better PFS (45.7%; 95% CI, 20.3–68.0%) (*p* = 0.013) than those with aGvHD (19.7%; 95% CI, 5.5–40.3%) (Figure 6C). Patients who experienced cGvHD had a better PFS (46.1%; 95% CI, 21.0–68.0%) than those without cGvHD (23.7%; 95% CI, 6.8–46.3%) (*p* = 0.016) (Figure 6D).

### DLI and clinical outcome

All 13 patients received DLI after HSCT, including nine patients with prophylactic and four patients with therapeutic DLI. The four patients received therapeutic DLI following a salvaged chemotherapy after relapse and all died of relapse. Among the nine patients who received DLI to prevent relapse, four died of relapse, two from NRM, and two were alive until the last 3-year follow-up since HSCT. However, given the heterogeneity and limited number of DLI infusion, a univariate analysis of the impact of DLI was not available in this study.

### Univariate analysis of HSCT outcome

A univariate analysis was performed and summarized in Table 2; the analysis was mainly based on donor gender, recipient gender, age, diagnosis, cytogenetic risk group, disease status at HSCT, EMD, BM blasts, and GvHD. For relapse, age > 10 yeas (*p* = 0.035), a diagnosis of AML (*p* = 0.057), female donors (*p* = 0.038), and cGvHD (*p* = 0.031) were considered as favorable factors. However, female gender (*p* = 0.058), EMD (*p* = 0.003), and aGvHD (*p* = 0.066) were all adverse factors influencing NRM. Taken together, the intermediate risk group (*p* = 0.017), the absence of EMD (*p* = 0.012), the absence of aGvHD (*p* = 0.013), cGvHD (*p* = 0.016), and female donors (*p* = 0.027) were significant favorable factors influencing PFS.

**Table 2:** Univariate analysis of outcome of HSCT

Results at 2y	N	Rel. (%)	*p* value	NRM (%)	*p* value	PFS (%)	*p* value
Gender			0.504		0.058		0.540
Male	29	41.9		11.3		46.8	
Female	15	38.7		35.9		25.4	
Age			0.035		0.232		0.170
≤ 10 y/o	14	57.1		7.1		35.8	
> 10 y/o	30	35.5		26.3		38.2	
Diagnosis			0.057		0.897		0.344
AML	28	28.9		19.2		51.9	
ALL	13	67.3		15.4		17.3	
MPAL	3	66.7		33.3		0.00	
Risk Group			0.149		0.712		0.017
Adverse Risk	21	59.9		25.4		14.7	
Intermediate Risk	9	14.3		NA		71.4	
Favorable Risk	14	33.1		22.9		44.0	
EMD			0.792		0.003		0.012
EMD +	9	36.1		63.9		0.00	
EMD –	35	43.6		9.1		47.3	
Status at HSCT			0.643		0.484		0.372
PIF	18	40.2		17.6		42.2	
Late Rel/Ref	9	77.8		NA		22.2	
Early Rel/Ref	17	29.4		31.4		39.2	
BM blasts			0.225		0.991		0.158
≤ 25%	16	40.5		20.3		39.2	
> 25%	28	48.0		19.2		32.8	
Donor Gender			0.038		0.672		0.027
Male	31	54.6		21.1		24.3	
Female	13	21.0		16.1		62.9	
Conditioning			0.295		0.757		0.492
TBI	31	41.3		20.5		38.2	
Bu	13	51.3		18.0		30.8	
Chemotherapy ^*^			0.089		0.269		0.621
Null	12	54.2		8.3		37.5	
FLAG	24	47.3		22.7		30.0	
CLAG	8	12.5		31.3		56.3	
Acute GvHD			0.275		0.066		0.013
aGvHD	19	48.7		31.6		19.7	
No aGvHD	25	39.1		10.0		51.0	
Chronic GvHD			0.031		0.880		0.016
cGvHD	20	32.4		21.2		46.4	
No cGvHD	24	52.3		18.1		29.6	

Abbreviations: y/o,years old; AML, acute myeloid leukemia; ALL, acute lymphocytic leukemia; MPAL, mixed phenotype acute leukemia; PIF, primary induction failure; EMD, extramedullary disease; Rel, relapse; BM, bone marrow; aGvHD, acute graft versus host disease; cGvHD, chronic graft versus host disease; DLI, donor lymphocyte infusion; NRM, non-relapse mortality; PFS, progression free disease; CLAG, cladribine, cytarabine, granulocyte colony stimulating factor; FLAG, fludarabine, cytarabine, granulocyte colony stimulating factor

^*^chemotherapy prior to conditioning.

### Multivariate analysis of HSCT outcome

Tables 3 and 4 showed the results of multivariate analysis, which failed to reveal any covariate associated with NRM. Firstly, a diagnosis was not associated with neither relapse nor PFS statistically. Male recipients were associated with inferior outcomes of relapse (hazard ratio [HR] = 3.98, *p* = 0.011) but not with PFS or OS. Adolescent patients only showed a superior outcome in relapse (HR = 0.25, *p* = 0.014) but not statistically in PFS or OS, indicating a probable increase hazard of NRM in this cohort. A diagnosis of ALL (HR = 4.31, *p* = 0.025) and the status of R/Ref (HR = 2.96, *p* = 0.060) at HSCT were linked to higher risks of relapse; however, these factors did not significantly influence patient’s survival. In addition, the intermediate-adverse risk group was associated with a worse outcome in relapse only (HR = 5.57, *p* = 0.031). Acute GvHD (HR = 12.85, *p* > 0.001) and EMD (HR = 11.21, *p* = 0.001) showed inferior PFS. Most importantly, chronic GvHD and the choice of a female donor were associated with superior PFS and OS as well as lower relapse.

**Table 3:** Multivariate analysis of relapse and NRM

Covariates	Relapse			NRM	
	SHR	95% CI (%)	SHR		95% CI (%)
Gender	*P* = 0.011		Not Entered		
Female	1.00	1.36–11.58			
Male	3.98				
Age	*P* = 0.014		*P* = 0.207		
≤ 10 y/o	1.00	0.08–0.75	1.00		0.44–46.41
> 10 y/o	0.25		4.50		
Diagnosis	*P* = 0.025		Not Entered		
AML	1.00	1.20–15.55			
ALL	4.31				
Risk Group	*P* = 0.031		*P* = 0.717		
Favorable	1.00	1.17–26.51	1.00		0.12–4.42
Intermediate/Adverse	5.57		0.71		
EMD	*P* = 0.218		*P* = 0.192		
EMD (–)	1.00	0.57–11.76	1.00		0.51–28.00
EMD (+)	2.59		3.79		
Status at HSCT	*P* = 0.060		*P* = 0.976		
P/Ref	1.00	0.96–9.15	1.00		0.07–13.72
R/Ref	2.96		0.96		
BM blasts	*P* = 0.349		*P* = 0.971		
≤ 25%	1.00	0.08–2.40	1.00		0.13–7.34
> 25%	0.45		0.96		
Donor Gender	*P* = 0.045		*P* = 0.618		
Male	1.00	0.00–0.92	1.00		0.09–4.20
Female	0.04		0.61		
Conditioning	*P* = 0.181		*P* = 0.359		
TBI	1.00	0.64–10.26	1.00		0.26–40.10
Bu	2.57		3.25		
Acute GvHD	*P* = 0.483		*P* = 0.119		
aGvHD (–)	1.00	0.40–7.07	1.00		0.60–82.20
aGvHD (+)	1.67		7.05		
Chronic GvHD	*P* = 0.008		*P* = 0.862		
cGvHD (–)	1.00	0.00–0.49	1.00		0.11–6.32
cGvHD (+)	0.06		0.84		

Abbreviations: y/o, years old; AML, acute myeloid leukemia; ALL, acute lymphocytic leukemia; MPAL, mixed phenotype acute leukemia; CNS, central nerves system; P/Ref, primary/refractory; Rel/Ref, relapsed/refractory; EMD, extramedullary disease; BM, bone marrow; aGvHD, acute graft versus host disease; cGvHD, chronic graft versus host disease; PFS, progression free disease; HR, hazard ratio; CI, confidence interval

**Table 4:** Multivariate analysis of PFS and OS

Covariates	PFS			OS	
	SHR	95% CI (%)	SHR		95% CI (%)
Gender	*P* = 0.217		*P* = 0.087		
Female	1.00	0.16–1.51	1.00		0.11–1.16
Male	0.50		0.36		
Age	*P* = 0.279		*P* = 0.314		
≤ 10 y/o	1.00	0.17–1.68	1.00		0.15–1.82
> 10 y/o	0.53		0.53		
Diagnosis	*P* = 0.200		*P* = 0.262		
AML	1.00	0.67–6.95	1.00		0.58–7.59
ALL	2.15		2.09		
Risk Group	*P* = 0.080		*P* = 0.264		
Favorable	1.00	0.86–13.63	1.00		0.53–10.25
Intermediate/Adverse	3.43		2.33		
EMD	*P* = 0.001		*P* = 0.001		
EMD (–)	1.00	2.83–44.35	1.00		2.40–33.79
EMD (+)	11.21		9.00		
Status at HSCT	*P* = 0.113		*P* = 0.281		
P/Ref	1.00	0.76–12.77	1.00		0.52–9.55
R/Ref	3.13		2.23		
BM blasts	*P* = 0.764		*P* = 0.759		
≤ 25%	1.00	0.16–3.90	1.00		0.13–4.37
> 25%	0.78		0.76		
Donor Gender	*P* < 0.001		*P* = 0.001		
Male	1.00	0.01–0.29	1.00		0.01–0.32
Female	0.06		0.06		
Conditioning	*P* = 0.102		*P* = 0.231		
TBI	1.00	0.78–14.89	1.00		0.55–11.94
Bu	3.42		2.56		
Acute GvHD	*P* < 0.001		*P* < 0.001		
aGvHD (–)	1.00	3.42–48.29	1.00		2.80–36.76
aGvHD (+)	12.85		10.15		
Chronic GvHD	*P* < 0.001		*P* = 0.002		
cGvHD (–)	1.00	0.02–0.30	1.00		0.03–0.45
cGvHD (+)	0.07		0.11		

Abbreviations: y/o, years old; AML, acute myeloid leukemia; ALL, acute lymphocytic leukemia; MPAL, mixed phenotype acute leukemia; CNS, central nerves system; P/Ref, primary/refractory; Rel/Ref, relapsed/refractory; EMD, extramedullary disease; BM, bone marrow; aGvHD, acute graft versus host disease; cGvHD, chronic graft versus host disease; PFS, progression free disease; HR, hazard ratio; CI, confidence interval.

## DISCUSSION

At present, few reports have been published specifically focusing on the salvaged HSCT for P/Ref and R/Ref acute leukemia in pediatric patients [8, 10, 12, 26–28]. This study presents the results from a relatively large, single-center Chinese population. The patients repeatedly failed to achieve CR prior to HSCT, yet achieved a stable engraftment and CR after HSCT following a sequential intensified conditioning. The estimated 2-year OS and PFS were 34.3% and 33.6%, respectively, and were largely comparable with large, registry-based studies and in accordance with those outcome from several studies based on adult patients in a similar situation [22–24, 27, 28]. In addition, after stratification by several covariates, this result can be deduced and compared with other published data, suggesting that the efficiency of salvaged allo-HSCT is not certain in patients with P/Ref and R/Ref pediatric acute leukemia who are not in remission.

Different with outcome from reports on *de novo* acute leukemia, children younger than 10 years did not show significantly superior outcomes compared with adolescents. It is well accepted that age is a strong indicator of prognosis in pediatric ALL cases, although not so definite in pediatric AML compared with ALL [14, 29]. However, the conclusion of this study is totally contradictory. An increased relapse hazard was observed in patients younger than 10 years, but this impact on PFS or OS was not significant. Firstly, less relapse in *de novo* pediatric acute leukemia cases is probably attributed largely to the underlying favorable biological features of leukemia frequently harbored in patients younger than 10 years [29]. However, it is reasonable to infer that the protective effect originating from cytogenetic features is not adequate or that it does not even exist in these subjects with P/Ref or R/Ref disease when compared with those without disease recurrence. Besides, another explanation would be an increased intensified chemotherapy-related NRM in adolescent patients due to comorbidities and advanced age or elevated relapse after being precluded from therapeutic protocol specifically for pediatric acute leukemia cases. Since all patients received the salvaged protocol of equivalent intensity and had similar performance status, this impact was re-balanced.

EMD prior to HSCT is more linked to increased NRM not relapse, which led to inferior PFS and OS. Isolated BM relapse has been reported to be associated with worse survival than both BM and EM relapse [30, 31]. However, the latter subgroup in our study showed significantly increased NRM (HR 3.79, *P* = 0.192), resulting in worse PFS and OS. The most reasonable explanation for this would be the toxicity caused by local irradiation and TBI-based conditioning regimen that is preferably administered to patients with EMD prior to HSCT and poor performance related to previous heavy treatment. In addition, given that EMD along with active BM relapse is very challenging to manage, as regular local irradiation to EMD is not available, lethal aGvHD intentionally induced by relatively rapid IST withdrawal with the purpose of reducing relapse originating from EMD was more frequently observed in this cohort. Among the 8 cases of deaths in 9 patients with EMD, only 3 were due to relapse; the remaining 5 were caused by NRM. Univariate analysis also showed that NRM rate of the EMD-positive cohort was as high as 63.0% compared with only 9.1% in the negative subgroup (*p* = 0.003). Thus, optimized supportive care and EMD management protocol are still needed.

In accordance to current consensus partially, HSCT using a female donor resulted in a remarkably lower relapse rate but higher survival rate in this study. Several studies had concluded that the use of a female donor, especially when the recipient is a male, was associated with reduced relapse rates [5, 32–34]. First, the major explanation for the lower relapse rates would be the inherent information variance caused by sex chromosomes. Moreover, results from our study definitely proved this phenomenon. Meanwhile, several other institutes also reported that a female donor would not be an appropriate choice based on the fact that the use of female donors was associated with increased incidence of NRM, which could counterbalance the positive impact on relapse rates [35]. Actually, this dispute could be well resolved by classifying patients in terms of disease risk and status prior to HSCT.

More interestingly, NRM rates in patients undergoing HSCT with a female donor were not increased and the hazard was lower compared with that in cases using a male donor. The most probable explanation would be the combination of donor-recipient. It is well accepted that fetal micro-chimerism is helpful in increasing survival rates in pediatric HSCT cases [36]. Besides, HSCT using matched related donors showed superior NRM compared with that using haplo-identical donors. In our cohort, among a total of 13 patients undergoing HSCT with female donors, 5 received grafts from their mothers and another 5 received grafts from matched related donors; only 3 received grafts from haplo-identical donors. Additionally, another less possible reason may be the experience of BMT physicians in our institute and special and intense care for those recipients with female donors throughout the whole process of HSCT.

Of importance, we find that a BM blast ratio of over 25% at the time of HSCT is not an independent adverse prognostic factor in this study. Unlike the results noted in our study, almost all the studies universally point out that BM blast value of over 25% led to worse outcomes [8, 9, 21, 31, 37]. One study from several Japanese institutes disclosed that the 5-year disease free survival rate for refractory and relapsed pediatric acute leukemia cases with BM blast values of more than 25% was 19.3 whereas that for those with lower tumor burden was 35.0% (P = 0.097) [38]. Additionally, most of the BMT institutes do not recommend performing allo-HSCT for such patients [9, 21]. However, we did not observe a statistical significance either by univariate analysis or by multivariate analysis with the stepwise regression method. Firstly, after co-variate analysis of cGvHD and donor gender stepwise into the regression model, relapse HR in the subgroup with BM blast values of over 25% decreased to 0.45 from 1.69. This probably suggests that the choice of female donors and induction of cGvHD can significantly reduce relapse hazard after allo-HSCT. Another explanation would be that pediatric acute leukemic cells with active proliferation show better response to chemotherapy due to the larger proportion of cells in the G2 and M phase than those with low proliferation activity [39]. In this cohort, we found a significantly less P/Ref value in the subgroup with a high tumor burden (11/16 versus 7/28, *p* = 0.005). In addition, the powerful sequential intensified conditioning regimen diminishes the impact of different levels of tumor burden on disease recurrence.

Several studies have proven that aGvHD predicted remission in the subjects with refractory disease after allo-HSCT and that it is associated with improved outcomes [12]. However, in our experience, aGvHD was an independent factor predicting inferior PFS and it was associated with a high incidence of relapse. Our results showed that the estimated 100-day cumulative incidence of aGvHD was 43.6%, which is almost 2 times higher than that in cases of conventional HSCT for regular patients in CR. This finding was likely induced by the robust conditioning and rapid withdrawal of IST, and less possibly by prophylactic DLI. Two factors might have contributed to the increased NRM by aGvHD. First, as common sense, aGvHD requires long-term use of high-dose corticosteroids and delayed withdrawal of IST, which negatively impact disease control, especially in our cohort with active disease before HSCT. Thus, delayed immune reconstitution as a result of all aGvHD interventions mentioned above would lead to increased risk of disease recurrence. Second, lethal complications including organ failure, opportunistic infection, and hemorrhage are more frequently observed in patients with aGvHD compared to those undergoing the regular HSCT.

Multiple studies with HSCT candidates in remission and adults not in remission have indicated that cGvHD is a strong predictor of favorable outcomes [24, 40–42]. In agreement, both univariate and multivariate analysis in our study concluded that cGvHD was a favorable prognostic factor in terms of PFS and relapse. It is reasonable to speculate that cGvHD was accompanied by robust graft versus leukemia (GvL) effects, thereby preventing the recurrence of disease in patients with active leukemia. Thus, introduction of cGvHD may be an appealing strategy of therapy during the long-term follow-up period. However, several studies did not conclude that cGvHD was associated with improved survival [12, 43]. According to the analysis performed in these studies, this may be explained by the fact that more grafts with T-cell-depletion were used in that cohort impairing the graft versus leukemia (GvL) effect. Of note, the incidence of cGvHD in our study was much higher. The estimated 1-year cumulative incidence of cGvHD was 45.5% (95% CI 30.5–59.3%) while the values in the pediatric population depend on several variables and can range from as low as 6% in matched sibling cord blood transplantation cases to as high as 65% in matched unrelated donor (MUD) PBSC transplantation cases. We speculated that rapid IST withdrawal, enhanced conditioning regimen and prophylactic DLI all contributed to this outcome.

OS stratified by the diagnosis showed that refractory AML had better survival rates than ALL, while MPL had the worst survival rates as it lacked a plateau in OS. Relapse is a major challenge in HSCT for refractory ALL and MPL. Our findings agree with the conventional concept of survival among HSCT patients. Despite the use of minimal residual disease (MRD)-guided prophylactic DLI, the survival data did not favor the DLI group eventually. The most probable outcome of DLI in these patients was uncontrollable aGvHD without the interference from some robust GvL effects. Further, adverse complications would subject these patients to a high risk of NRM in addition to the relapsed disease. Thus, considerable future research is needed to solve the therapeutic challenges noted for patients with refractory ALL or MPL.

Admittedly, our study has a few limitations. First, it was a retrospective analysis which may bear an innate selection bias. This has been partly addressed by the careful selection of high quality data, directly from the investigators rather than from the registries. Further, our results were limited by the insufficient number of cases with ALL and MPL. The conclusion of our study may need validation from an independent, larger study with more ALL and MPL patients.

In conclusion, in this study, the estimated 2-year OS and PFS rates were 34.3% and 33.6%, respectively. Moreover, our analysis suggested that cGvHD with female donors improved PFS significantly, whereas EMD and aGvHD predicted poor survival. Moreover, male recipients, age younger than 10 years, ALL diagnosis, and being in an intermediate-adverse cytogenetic risk group were associated with increased relapse rates. These data suggest that pediatric patients with refractory acute leukemia probably benefit from salvaged allo-HSCT not in remission.

## MATERIALS AND METHODS

### Patients and donors

Pediatric patients aged 18 and younger with P/Ref and R/Ref AML, ALL, or mixed phenotype acute leukemia (MPAL) who received their first allo-HSCT at the department of hematology, China Aerospace Central Hospital, between May 2012 and December 2016 were included in this retrospective study. Patients with a diagnosis of FAB M3 or juvenile myelomonocytic leukemia were excluded from this study. The characteristics of all patients were summarized in Table 5. Briefly, 29 (65.9%) patients were male, and 15 (34.1%) were female. The median age at transplantation was 8 years with a range from 1 to 18 years. The diagnosis included 28 AML, 13 ALL, and 3 MPAL. Among the study population, 18 of them had P/Ref and 26 had R/Ref prior to transplantation. In the AML subgroup, two patients had secondary AML with a history of aplastic anemia (AA) and one was on the blast phase of chronic myeloid leukemia (CML). Of the 13 ALL patients with ALL, 9 of them had B-ALL and 4 had T-ALL. After reviewing the cytogenetic risks at diagnosis, we categorized the patients into three groups, favorable (*n* = 14), mediate (*n* = 9), and adverse (*n* = 21), following the 2008 WHO classification with the recommendation for pediatric leukemia [21]. Central nervous system (CNS) involvement before HSCT was observed in 11 patients, and the development of extramedullary disease (EMD) in sites other than the CNS was seen in another 9 patients. In the R/Ref group, 19 patients who had refractory relapse disease experienced one relapse, 6 patients had two relapses, and 1 patient had three relapses. Of note, 10 patients with BM blasts of 50% to 75% and 14 patients with blasts over 75% initialed the HSCT conditioning procedure. All patients were followed up until May 2017. This study was approved by the institutional review board of the Chinese Aerospace Central Hospital. All procedures were carried out according to the principles of the Declaration of Helsinki. A written informed consent was obtained from all patients enrolled in this study.

**Table 5:** Patients’ characteristic who underwent HSCT

Variables	
Age at HSCT, Median (Range)	8 (Range 1–18)
Gender, n (%)	
Male	29 (65.9)
Female	15 (34.1)
Types of leukemia, n (%)	
AML	28 (63.6)
ALL	13 (29.5)
MPAL	3 (6.8)
Disease status, n (%)	
P/Ref	18 (40.9)
Early R/Ref	17 (38.6)
Late R/Ref	9 (20.5)
Bone marrow blasts, Median (Range)	58.1% (6.0–98.0)
Extramedullary involvement at allo-HSCT, n (%)	
Yes	9 (20.5)
No	35 (79.5)
CNS involvement at allo-HSCT, n (%)	
Yse	11 (25.0)
No	33 (75.0)
Cytogenetics, n (%)	
Favorable risk	9 (20.5)
Mediate risk	8 (18.2)
Adverse risk	27 (61.3)
No. of chemotherapy lines, n (%)	
≤ 3	23 (52.3)
> 3	21 (47.7)
No. of chemotherapy cycles, n (%)	
≤ 8	26 (31.8)
> 8	18 (68.1)

Abbreviations: AML, acute myeloid leukemia; ALL, acute lymphocytic leukemia; MPAL, mixed phenotype acute leukemia; P/Ref, primary refractory; R/Ref, relapsed refractory; CNS, central nervous system;

### Definition of disease or survival status

Complete remission (CR) was defined as the return of normal hematopoiesis and the presence of less than 5% blasts in the BM, with no evidence of EMD. Relapsed leukemia was defined as the presence of over 5% blasts in the BM or proven EMD. P/Ref disease was defined as failed to achieve CR after two cycles of first-line chemotherapy or one cycle of intensified therapy. R/Ref was defined as failed to regain CR after two cycles of standard salvaged chemotherapy since relapse. The cytogenetic risk was evaluated according to the 2008 WHO classification, and specific pediatric recommendations were reviewed and confirmed for every patient [21].

Neutrophil engraftment was defined as the first day of 3 consecutive days with a neutrophil count over 0.5 × 10^9^/L. Platelet engraftment was defined as a platelet count higher than 20 × 10^9^/L with no transfusion for at least 7 days. The absence of hematopoietic recovery at day 60 and autologous hematopoietic reconstruction were considered as engraftment failure. Complete donor chimerism was defined as the presence of over 95% donor-originated cells, and mixed chimerism was defined as the detection of 10–95% donor-derived cells in the BM by DNA fingerprints of short tandem repeats (STRs). Poor graft function was diagnosed in patients with 2–3 lineages (Hb < 10 g/dL, neutrophil count < 1.0 × 10^9^/L, and platelet count < 30 × 10^9^/L) for at least 2 consecutive weeks beyond day +14 post-transplantation, with transfusion requirement, associated with hypoplastic-aplastic BM, in the presence of complete donor chimerism and in the absence of severe graft versus host disease (GvHD) and relapse.

Acute and chronic GvHD were scored according to the criteria proposed by the 1994 Consensus Conference on Acute GvHD and the NIH Consensus Development Project on Criteria for Clinical Trials in Chronic GvHD [44]. Overall survival (OS) was defined as the duration from the day of HSCT (0 day) to death of any cause. Progression-free survival (PFS) was defined as the time from transplantation to relapse or death of any cause.

### HLA typing and donor selecting

The HLA compatibility was tested by high-resolution typing of HLA-A, HLA-B, HLA-C, HLA-DR, and HLA-DQ. Matched-related donors were defined as the sibling donors with identical HLA typing, inheriting the same parental haplotypes as the recipients. Otherwise, the donors were called HLA haplo-identical donors. Unrelated donors were not included in the study due to the delayed excess of HSCT. The appropriate donor was first chosen from the matched-related donors; otherwise, haplo-identical donors were used. All recipients receiving haplo-identical HSCT should receive an additional umbilical cord blood (UCB) infusion except those with contraindications evaluated by physicians to enhance engraftment and reduce severe acute GvHD as previously reported [45]. UCB were selected based on the HLA typing and cell doses and infused at least 4 hours prior to BM infusion. The HLA loci of each UCB unit should be at least 3/6 matched with that of the recipient. However, in our institute, this protocol-proposed dose of UCB total nucleated cell (TNC) was modified into 1.0 × 10^7^/Kg at least 4 hours prior to BM infusion.

### Conditioning regimen and GvHD prophylaxis

All patients received either busulfan-based (Bu) or total body irradiation (TBI)-based myeloablative conditioning regimens. In general, myeloid leukemia patients received a Bu-based regimen, while those with lymphoid leukemia or EMD received a TBI-based regimen. These two regimens were administered as follows:

1) Bu given intravenously 0.8 mg/Kg/6 h (day –8 to day –6), CTX 1.8 g/m^2^ (day –5 and day –4), and Me-CCNU 250 mg (day -3)

2) TBI at a dose of 200 cGy for consequently six fractions (day –8 to day –6), CTX 1.8 g/m^2^ (day –5 and day –4), and Me-CCNU 250 mg (day –3)

**Figure 7:**
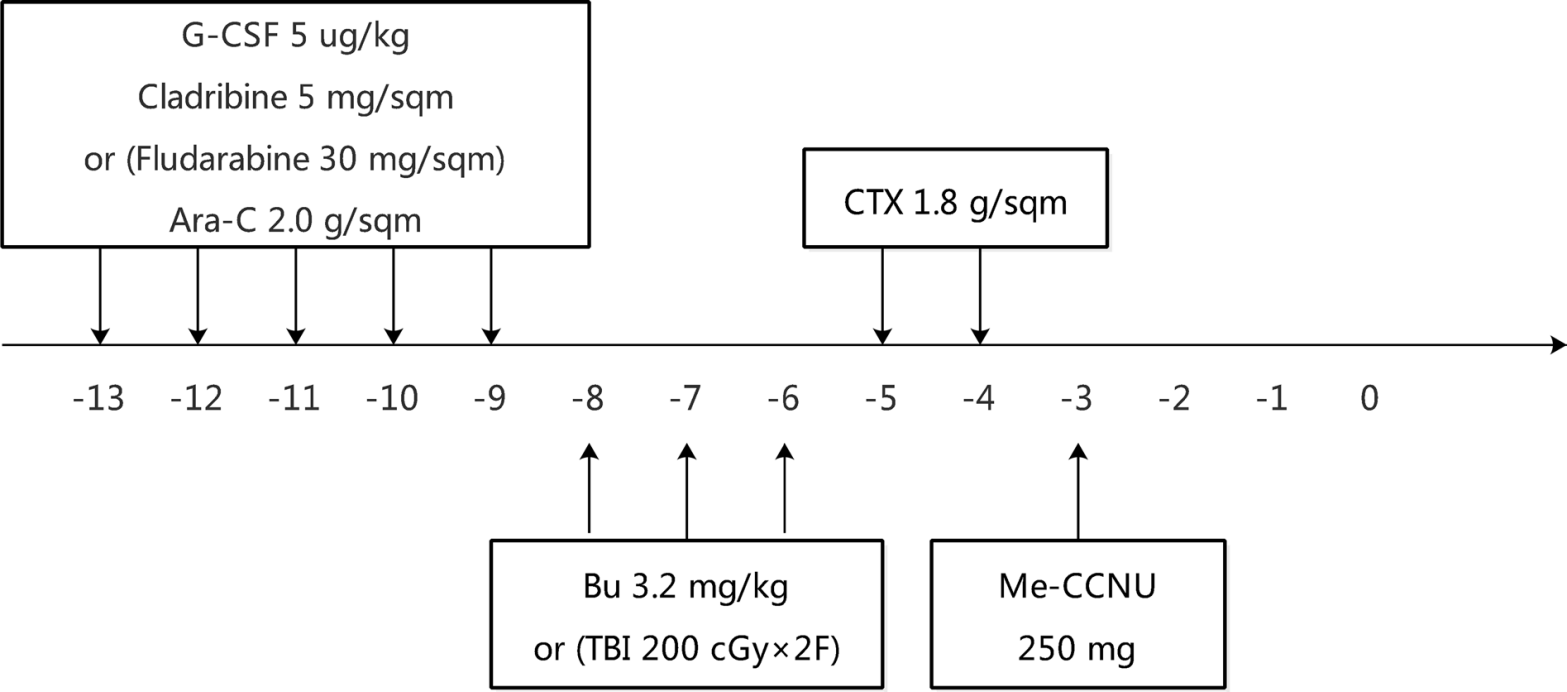
Design of conditioning regimens

An antithymocyte globulin (ATG, Fresenius) was infused at a total dose of 20–40 mg/Kg/day in 4 days from day –5 to day –2. Prior to the conditioning regimen, patients received FLAG (fludarabine 30 mg/m^2^ for 5 days, cytarabine 2 g/m^2^ for 5 days, and granulocyte colony-stimulating factor 5 ug/Kg for 5 days) or CLAG (cladribine 5 mg/m^2^ for 5 days, cytarabine 2 g/m^2^ for 5 days, and granulocyte colony-stimulating factor 5 ug/Kg for 5 days) as a cyto-reduction regimen to enhance the myeloablative effect (Figure 7). Either of these two chemotherapy regimens was advised by the attending physician, based on the previous exposure to fludarabine or cladribine. However, patients whose performance score is less than 75 are not recommended to receive cyto-reduction chemotherapy.

GvHD prophylaxis consisted of mycophenolate mofetil (MMF), cyclosporine A (CsA) and a short course of methotrexate (MTX) administered at days +1, +3, and +6, at a dose of 10 mg/m^2^.

Patients received BM and peripheral blood stem cells (PBSCs) from the same donor as the graft is infused at day 0 and 1, consequently. At day 0, at least 4 hours prior to haplo-identical BM infusion, patients received a single unit of UCB infusion from a third donor to enhance engraftment [45, 46]. However, in subjects with contraindications to UCB infusion, including those with uncontrolled hypertension, fever, low oxygen saturation, allergic event during conditioning, or history of immune-related organ impairment such as transfusion- or drug-related pneumonia, UCB infusion were omitted or canceled.

### Supportive care and post-HSCT management

All patients received HSCT in a high-efficiency laminar air flow room since the start of conditioning. A conventional gut decontamination protocol was used on all patients. The subcutaneous administration of G-CSF was not routinely used except for those patients with no evidence of neutrophil engraftment at day 10. A veno-occlusive disease was prevented with prostaglandin E1 (PGE1). All blood products were infused after irradiation and leukocyte depletion to maintain a hemoglobin level above 60 g/L and a platelet count over 20,000/uL.

After neutrophil recovery, bone marrow aspirations were repeated every 4 weeks for the first 6 months after transplant along with chimerism evaluation by STR analysis and every 3 months, thereafter, until at least 1 year post-transplantation. However, a temporary BM examination in case of suspicious disease progression was also considered. Cytomegalovirus (CMV) and Epstein-Barr virus (EBV) viremias were accessed by real-time PCR-based method twice a week. Ganciclovir or foscarnet was delivered as pre-emptive therapy if CMV DNA viremia was positive. EBV viremia was treated with upfront rituximab, an anti-human CD20 monoclonal antibody. For those patients with viremia refractory to conventional treatment, CMV- and/or EBV-specific cytotoxic lymphocytes infusion were considered.

Immunosuppressive therapy (IST) withdrawal started shortly after BM evaluation on +28 day after HSCT, in case of no overt acute GvHD. In brief, MMF was discontinued since +28 day after HSCT and the valley CsA serum concentrations were maintained at 200–250, 100–150, and lower than 100 ng/mL for the first 3 months after HSCT. CSA was stopped within the third month after HSCT for recipients without GvHD. Acute and chronic GvHD was scored according to the criteria proposed by the 1994 Consensus Conference on Acute GvHD and the NIH Consensus Development Project on Criteria for Clinical Trials in Chronic GvHD.

Donor lymphocyte infusion (DLI) was classified into two categories: therapeutic or prophylactic DLI. Therapeutic DLI was given to patients with disease progression at minimal residual disease (MRD) level or following a salvaged chemotherapy for patients with hematological relapse. By contrast, the latter was performed to subjects with stable MRD without aGvHD in the first 3 months or without cGvHD within the first 6 months. Each infusion of DLI product consisted of a median dose of mono-nuclear cells (MNCs) of 1.0 × 10^8^/Kg. Patients receiving DLI from an HLA-matched related or haplo-identical donor did not receive any agent to prevent GvHD after each infusion.

### Statistical analysis

The data were collected from the institutional database and verified by the primary investigators and staff of the HSCT team. The data were checked for consistency and analyzed using the Stata Release 14 (StataCorp LLC, TX) and SPSS version 23.0 (SPSS Inc, IL). The last follow-up was done on May 31, 2017. The descriptive analysis was reported as median and range. The Kaplan-Meier estimators and confidence intervals were used for PFS and OS. The log-rank test was used for comparison. The cumulative incidence rates of relapse and non-relapse mortality (NRM) were estimated, allowing for competing risks using the Fine-Gray method [47].

The univariate and multivariate analyses of the significance of covariates affecting PFS or relapse and NRM were determined using the Cox proportional hazard model or Fine-Gray regression model, respectively [48]. The parameters calculated in the univariate analysis included age, gender, donor gender, diagnosis, cytogenetic risk classification at diagnosis, pre-HSCT chemotherapy cycles, duration of first remission, disease status and BM blast burden prior to HSCT, and acute and chronic GvHD. The confidence interval was reported at 95%, and the statistical analysis was performed at the 0.05 level (two sided).
